# Rapid cycle deliberate practice vs. traditional simulation in a resource-limited setting

**DOI:** 10.1186/s12909-019-1742-4

**Published:** 2019-08-22

**Authors:** Samantha L. Rosman, Rosine Nyirasafari, Hippolyte Muhire Bwiza, Christian Umuhoza, Elizabeth A. Camp, Debra L. Weiner, Marideth C. Rus

**Affiliations:** 1 Department of Pediatrics, Harvard Medical School, Division of Emergency Medicine/Harvard Medical School, 300 Longwood Ave, Boston, MA 02130 USA; 2 Department of Pediatrics, University of Rwanda, Kigali, Rwanda; 3 Department of Pediatrics, Section of Pediatric Emergency Medicine, Baylor College of Medicine/ Texas Children’s Hospital, Houston, TX USA

**Keywords:** Rapid cycle deliberate practice, Simulation, Pediatrics, Low-fidelity, Resource-limited, Resuscitation, Curriculum, Resident education, Africa

## Abstract

**Background:**

We sought to develop a low-fidelity simulation-based curriculum for pediatric residents in Rwanda utilizing either rapid cycle deliberate practice (RCDP) or traditional debriefing, and to determine whether RCDP leads to greater improvement in simulation-based performance and in resident confidence compared with traditional debriefing.

**Methods:**

Pediatric residents at the Centre Hospitalier Universitaire de Kigali (CHUK) were randomly assigned to RCDP or traditional simulation and completed a 6 month-long simulation-based curriculum designed to improve pediatric resuscitation skills. Pre- and post- performance was assessed using a modified version of the Simulation Team Assessment Tool (STAT). Each video-taped simulation was reviewed by two investigators and inter-rater reliability was assessed. Self-confidence in resuscitation, pre- and post-simulation, was assessed by Likert scale survey. Analyses were conducted using parametric and non-parametric testing, ANCOVA and intra-class correlation coefficients (ICC).

**Results:**

There was a 21% increase in pre- to post-test performance in both groups (*p* < 0.001), but no difference between groups (mean difference − 0.003%; p 0.94). Inter-rater reliability was exceptional with both pre and post ICCs ≥0.95 (p < 0.001). Overall, self-confidence scores improved from pre to post (24.0 vs. 30.0 respectively, p < 0.001), however, the there was no difference between the RCDP and traditional groups.

**Conclusions:**

Completion of a six-month low-fidelity simulation-based curriculum for pediatric residents in Rwanda led to statistically significant improvement in performance on a simulated resuscitation. RCDP and traditional low-fidelity simulation-based instruction may both be valuable tools to improve resuscitation skills in pediatric residents in resource-limited settings.

**Electronic supplementary material:**

The online version of this article (10.1186/s12909-019-1742-4) contains supplementary material, which is available to authorized users.

## Background

Children frequently become critically ill, especially in resource-limited settings, and require rapid assessment and resuscitation. Expeditious recognition and management of serious conditions such as respiratory distress, seizures, altered mental status, shock, and cardiac arrest is critical [1–4].

Simulation has been used in many settings to teach students and residents resuscitation skills. Simulation-based educational interventions have been shown to improve resident knowledge and performance in studies assessing simulation for neonatal and pediatric resuscitations [5–7], pediatric airway management [8], and time to initiation of CPR [9]. While these studies were conducted on high-fidelity simulators, there is also evidence that low-fidelity simulation can be effective, and that the transfer of learning is not dependent on the simulator fidelity [10]. However, there is little research to evaluate the use of low-fidelity simulation to teach complex scenarios such as management of respiratory failure, seizures, and cardiopulmonary resuscitation, particularly in pediatrics.

There are a number of studies of simulation curricula showing improved care delivery skills among physicians, nurses and allied health professionals in low-resource settings [11–14] but none that we know of for pediatrics residents. Improving the resuscitation skills of pediatric residents in a global health setting where they are frequently tasked with treating critically ill children has the potential to decrease in-hospital pediatric mortality rates significantly [4, 15].

Rapid Cycle Deliberate Practice is a simulation training technique in which a simulation is segmented into less complex parts and each individual part or skill is repeated with rapid debriefing cycles until expert performance is obtained, at which point the scenario is allowed to advance to the next step or level of difficulty [16, 17]. It allows for feedback to be provided and areas for improvement to be identified as mistakes are made, rather than reflectively at the end of the scenario, and allows the level of difficulty to adjust to the competency of the learner. Studies have shown significant improvement in learner skills using RCDP [16, 18, 19]. Traditional simulation debriefing typically occurs at the end of the resuscitation with a longer period of time to reflect on performance and develop plans for modifications to use during subsequent resuscitations [20].

The aim of this study to compare self-confidence in resuscitation skills and technical performance in simulated pediatric resuscitations of residents trained with a simulation-based curriculum utilizing traditional simulation debriefing to those trained utilizing RCDP.

## Methods

This study was conducted at Centre Hospitalier Universitaire de Kigali (CHUK) [21]. CHUK is the main academic and referral hospital for the country of Rwanda that serves as one of two main teaching hospitals for the University of Rwanda. In 2015, the Pediatric Department admitted 2242 patients with a mortality rate of 10.4%. There had been a traditional simulation program that most post-graduate year (PGY) 2–4 residents had participated in prior to the start of the study without a formal curriculum or case set. RCDP had not been utilized previously.

All Rwandan pediatric residents at CHUK who consented to participate in the study were eligible. While participation in the simulation sessions was a requirement of the resident curriculum, residents were able to decide whether or not to allow their data to be analyzed for purposes of this study. We enrolled a total of 51 pediatrics residents out of a total of 52 eligible residents with one resident declining participation. Using the expected sample size of 52 (alpha = 0.05; power = 0.80), the smallest detectable mean difference was determined to be 0.09, with a corresponding experimental group mean of 0.30.

Pediatric residents were divided by post-graduate year group and then randomized within each group using a random number generator (randomization.com generator #1) assigning each resident to one of two simulation curriculum groups (RCDP or traditional simulation). Given odd numbers within year groups we ended up with slightly imbalanced groups with 27 assigned to RCDP and 24 assigned to traditional.

Residents completed a six-month long simulation-based curriculum designed to improve pediatric resuscitation skills. Four cases were developed for each of three categories: shock, respiratory failure, and cardiac arrest. Between January and June 2015 residents participated in zero to five 1-h simulation sessions over the 6-month period with an average of 2.7 sessions per resident. Simulations teaching sessions were held 2–3 times per week to enable participation of all residents rotating at CHUK but due to rotation schedules there were 3 residents who did not rotate at CHUK at all during our curriculum period. We attempted to change case-type by month but due to resident rotation schedules each resident completed different cases within those categories and did not necessarily complete a case in each category.

All simulation teaching sessions for both the RCDP and traditional were conducted by the same instructor board certified in the U.S. in Pediatric Emergency Medicine (SLR) in partnership with a Rwandan pediatric senior resident (HMB, CU or RN). Residents received instruction for cases based on the assigned group. For RCDP cases, residents participated in several rounds, which each round becoming more complex than the previous. RCDP sessions were paused after each round to allow immediate feedback and repeat performance before continuing to the next round. For traditional debriefing cases, residents completed the full case before debriefing. The final, most complex round of the RCDP case was used, with minimal modifications, as the full case for the traditional group. (see Additional Items Additional files 3 and 4 for sample RCDP and traditional cases).

Our study used low- to mid-fidelity simulation utilizing a Simulaids® Pediatric ALS Trainer mannequin and the Laederal® ALS Baby mannequin. We had basic intubation equipment and a bag mask ventilator. We had AED pads but no AED simulator. We did not have IVs or IOs to place so obtaining vascular access was verbalized with IO technique specifically verbalized if this was the chosen route of access. We used empty syringes to simulate medications and boluses. We used the SimMonitor ipad/iphone app which can be controlled via Bluetooth® to provide patient monitoring feedback.

Residents participated in a pre- and post-intervention simulation with performance evaluated using a modified version of the Simulation Team Assessment Tool (STAT), a tool designed to evaluate team performance in simulated pediatric resuscitations (Additional files 1 and 2) [22]. This tool was modified to reflect differences in resources in our setting, both in terms of supplies and team size. Our modified 65-item tool used a standardized grading schematic to assess the following domains: Basic (history, assessment, access, etc.), Airway & Breathing (assessment, airway and bag-mask ventilation, and intubation skills), Circulation (assessment, fluid resuscitation, CPR and arrhythmias), and Team Management Tasks. Each item received a score of 0–2, with the scores for each item added to determine the total score for each resident’s performance. Pre- and post-intervention resuscitation scenarios were videotaped to allow for scoring by an observer (MCR) who was blinded by group with the in-person unblinded observer (SLR) scores used only to assess for inter-observer reliability.

Residents also completed basic demographic information, a pre- and post-intervention subjective self-assessment of their resuscitation skills using a five point Likert scale ranging from 1 = not at all confident to 5 = very confident, I have mastered this skill and could teach it to others, and satisfaction with the simulation curriculum using a five point Likert scale ranging from 1 (not helpful) to 5 (exceptionally helpful).

This study was approved by the Boston Children’s Hospital, Baylor College of Medicine, and Centre Hospitalier Universitaire de Kigali Internal Review Boards.

### Statistical analysis

Continuous variable distributions were assessed to determine the appropriate statistical test for analysis. When assessing distribution for the demographic variables, both factors (years practiced and pediatric training in months) were found to be skewed and therefore non-parametric (Mann-Whitney) testing was utilized with median and interquartile ranges reported. For categorical variables, the Pearson Chi-Square test was used unless cell values were less than five, then the Fisher’s Exact Test was utilized. The distributions of the pre- and post-STAT grading scores were normally distributed as determined by the Shapiro-Wilk Test of Normality. Both pre- and post-evaluation scores were normally distributed therefore parametric testing (paired and independent t-test) was used for analysis. The groups mean and standard deviations were reported. In addition to the two tests, a univariate linear model was created to adjust for pre-test scores. Before the ANCOVA analysis was conducted, a pre-analysis with an interaction term was created to determine if the homogeneity of slopes assumption was violated. ANCOVA adjusted post evaluation means were used for differences among groups on their pre-evaluations, because these differences are likely to occur within groups.

To determine the inter-rater reliability of the STAT continuous score, an intra-class correlation coefficient (ICC) was calculated. The analysis parameters were a two-way mixed model for fixed raters with an absolute agreement between raters. An ICC ≥ 0.8 was considered an acceptable agreement.

Self-confidence was assessed during the pre- and post-intervention time frames in both groups. Non-parametric testing was utilized to analyze the ordinal data, the Sign test for matched pairs and the Mann-Whitney test for independent groups. Satisfaction with the curriculum was assessed in the post-intervention survey. Even though the satisfaction data was Likert-based, only 2 categories were selected by respondents so a chi-square, unadjusted odds ratio and a Mann-Whitney test were used.

Statistical significance was defined as *p*-value< 0.05 All analyses were conducted using the Statistical Package for Social Science, version 24 (IBM Corp., Armonk, NY).

## Results

Demographics were not significantly different in the RCDP and traditional simulation groups (Table 1). Due to technical issues with recording of simulation testing performance that prevented our blinded observer from rating all simulation testing sessions a total of 23 residents were used for final analysis with 13 in the traditional group and 20 in the RCDP group. Performance, as measured by the STAT score, increased significantly (*p* < 0.001) between pre-evaluation ($\overline{\mathrm{x}}=0.45\ \left(\pm 0.12\right)$) and post-evaluation ($\overline{\mathrm{x}}=0.67\ \left(\pm 0.12\right)$) for all residents as a group as well as by study group (Table 2) without significant difference in % change in STAT score between RCDP and traditional groups ($\overline{\mathrm{x}}=\hbox{-} 0.003\ \left(95\%\mathrm{CI}\hbox{-} 0.08-0.08\right)$; *p* = 0.94) (Table 3).

**Table 1:** Demographic Comparisons RDCP vs. Traditional Simulation Training Groups (*N* = 33)

	Traditional *N* = 13 (39.4%) N (%) or Median (IQR)	RCDP *N* = 20 (60.6%) N (%) or Median (IQR	p-value
Residency Level			
I	3 (23.1)	6 (30.0)	0.60
II	4 (30.8)	4 (20.0)	
III	6 (46.2)	8 (40.0)	
IV	0 (0.0)	2 (10.0)	
Years of Practice	2.0 (2.0, 3.50)	2.0 (1.50, 3.0)	0.23
Medical School			
UR	12 (92.3)	16 (80.0)	0.63^a^
Other	1 (7.7)	4 (20.0)	
Taken BLS			
No	2 (15.4)	8 (40.0)	0.25^a^
Yes	11 (84.6)	12 (60.0)	
Taken PALS			
No	7 (53.8)	8 (40.0)	0.44
Yes	6 (46.2)	12 (60.0)	
Taken ETAT			
No	3 (23.1)	2 (10.0)	0.36^a^
Yes	10 (76.9)	18 (90.0)	
ETAT Instructor			
No	11 (84.6)	19 (95.0)	0.55a
Yes	2 (15.4)	1 (5.0)	
PED Months			
0	6 (46.2)	8 (40.0)	0.63
1	5 (38.5)	6 (30.0)	
2	2 (15.4)	6 (30.0)	
Number of Resuscitations			
0–10	6 (46.2)	13 (68.4)	0.21
≥ 11	7 (53.8)	6 (31.6)	

*RDCP* rapid cycle deliberate practice, *UR* University of Rwanda, *BLS* basic life support, *PALS* pediatric advanced life support, *ETAT* emergency triage assessment and treatment, *PED* Pediatric Emergency Department

^a^ *p* value was calculated using Fisher’s Exact Test for tables with a cell value < 5

**Table 2:** Comparison of Paired Pre- and Post- STAT Scores Stratified by Study Groups (N = 33)

Groups	Pre-Evaluation Percent Mean (±SD)	Post-Evaluation Percent Mean (±SD)	Post-Pre Evaluation Percent Difference Mean	95% Confidence Interval	p-value^a^
Overall	0.45 (0.12)	0.67 (0.12)	0.21	0.17–0.25	< 0.001
Traditional	0.47 (0.10)	0.68 (0.11)	0.21	0.14–0.28	< 0.001
RCDP	0.44 (0.13)	0.66 (0.13)	0.21	0.16–0.26	< 0.001

^a^ P-value was calculated using the Paired t-Test

**Table 3:** Mean Percent Differences of Pre- and Post- STAT Test Scores between Study Groups (N = 33)

Traditional Group Percent Mean (±SD)	RCDP Percent Mean (±SD)	Mean Percent Difference	95% CI	p-value^a^
0.21 (0.11)	0.21 (0.11)	−0.003	− 0.08 – 0.08	0.94

*STAT* simulation team assessment tool

^a^ p-value was calculated using the Independent t-Test

Pre-analysis with an interaction term to determined that the homogeneity of slopes assumption was not violated with the resulting p-value not significant (p-value = 0.52),. Results from the ANCOVA analysis show that the population adjusted means are equal (F = 0.06; p-value = 0.81; partial eta squared = 0.002). With a partial eta square of 0.002, there is little to no relationship between study group and post-test when controlling for pre-test results.

Inter-rater reliability was excellent for pre- (ICC = 0.95 (95% CI 0.90–0.98); p-value< 0.001) and post- (ICC = 0.96 (95% CI 0.93–0.98); p-value< 0.001) evaluation scores.

All self-confidence scores either increased by one point or remained the same after the intervention. Overall and in the RCDP group, all self-confidence items and the total score were significantly increased from pre- to post-testing (Table 4). Within the traditional group, placing an IO line, bag valve mask (BVM) management and chest compressions were significantly increased in pre- vs. post-testing (Table 4). All total Likert scores significantly increased after the intervention, overall and in both study groups (Table 4). When comparisons in self-confidence were made between groups and stratified on pre- and post-testing, no significant differences were found (Table 5). However, there was a trend towards a greater improvement in the RCDP group with a 3-point increase from pre- to post-self-confidence in the traditional group and a 6-point increase in the RDCP group. Overall, there is a significant difference between pre- and post- self-evaluation scores, but the study groups had no effect on self-confidence.

**Table 4:** Comparison of Pre- and Post-Test Self-Confidence using Likert-Scale^c^ Paired Data

Confidence in ability to:	Pre-Test Median (IQR)	Post-Test Median (IQR)	p-value^a^
Overall (N = 33)			
Place IO line	3.0 (3.0, 4.0)	4.0 (3.50, 4.0)	< 0.001
Manage BVM ventilation	4.0 (3.50, 4.0)	4.0 (4.0, 5.0)	0.001
Intubate	3.0 (1.0, 3.0)	3.0 (3.0, 4.0)	< 0.001
Perform chest compressions	4.0 (3.0, 4.0)	5.0 (4.0, 5.0)	< 0.001
Evaluate a rhythm strip	3.0 (2.0, 3.0)	3.0 (3.0, 4.0)	< 0.001
Use an AED or defibrillator	2.0 (1.0, 3.0)	3.0 (2.0, 4.0)	< 0.001
Lead resuscitation team w/good pulse^b^	3.0 (3.0, 4.0)	4.0 (4.0, 4.0)	< 0.001
Lead resuscitation team w/cardiac arrest	3.0 (3.0, 4.0)	4.0 (3.0, 4.0)	< 0.001
Total Likert Score^b^	24.0 (21.0, 28.75)	30.0 (27.0, 34.0)	< 0.001
Traditional Group (n = 13)			
Place IO line	4.0 (3.0, 4.0)	4.0 (4.0, 4.50)	0.13
Manage BVM ventilation	4.0 (4.0, 4.0)	4.0 (4.0, 4.50)	0.25
Intubate	3.0 (2.0, 3.0)	4.0 (3.0, 4.0)	0.01
Perform chest compressions	4.0 (3.50, 4.0)	5.0 (4.0, 5.0)	0.07
Evaluate a rhythm strip	3.0 (2.0, 3.0)	3.0 (3.0, 4.0)	0.04
Use an AED or defibrillator	2.0 (1.50, 3.0)	3.0 (2.50, 4.0)	0.04
Lead resuscitation team w/good pulse^b^	3.50 (3.0, 4.0)	4.0 (4.0, 4.0)	0.02
Lead resuscitation team w/cardiac arrest	3.0 (2.50, 4.0)	4.0 (3.0, 4.0)	0.03
Total Likert Score^b^	26.0 (21.75, 28.75)	30.0 (27.25, 31.75)	< 0.001
RCDP Group (n = 20)			
Place IO line	3.0 (3.0, 4.0)	4.0 (3.0, 4.0)	0.01
Manage BVM ventilation	4.0 (3.0, 4.0)	4.50 (4.0, 5.0)	0.01
Intubate	2.50 (1.0, 3.0)	3.0 (2.25, 4.0)	< 0.001
Perform chest compressions	4.0 (3.0, 4.75)	5.0 (4.0, 5.0)	< 0.001
Evaluate a rhythm strip	2.0 (2.0, 3.0)	3.0 (2.25, 4.0)	< 0.001
Use an AED or defibrillator	2.0 (1.0, 2.75)	3.0 (2.0, 4.0)	0.003
Lead resuscitation team w/good pulse	3.0 (3.0, 4.0)	4.0 (3.25, 4.75)	0.002
Lead resuscitation team w/cardiac arrest	3.0 (3.0, 3.75)	4.0 (3.0, 4.0)	0.01
Total Likert Score	23.0 (20.25, 28.75)	29.0 (26.25, 34.0)	< 0.001

^a^ p-value was calculated using the Sign Test

^b^ One value is missing for this variable

^c^ Likert-scale: 1 = not at all confident; 2 = slightly confident, I am able to initiate the skill but would prefer assistance or direction; 3 = somewhat confident, I am able to perform the skill well but would prefer some assistance or direction; 4 = confident, I am able to perform the skill well without assistance; and 5 = very confident, I have mastered this skill and could teach it to others

**Table 5:** Comparison of Traditional and RCDP Self-Confidence using Pre- and Post-Test Likert Scale ^c^

Confidence in ability to:	Traditional Group N = 13 Median (IQR)	RCDP Group N = 20 Median (IQR)	p-value^a^
Pre-Test			
Place IO line	4.0 (3.0, 4.0)	3.0 (3.0, 4.0)	0.14
Manage BVM ventilation	4.0 (4.0, 4.0)	4.0 (3.0, 4.0)	0.40
Intubate	3.0 (2.0, 3.0)	2.50 (1.0, 3.0)	0.28
Perform chest compressions	4.0 (3.50, 4.0)	4.0 (3.0, 4.75)	0.97
Evaluate a rhythm strip	3.0 (2.0, 3.0)	2.0 (2.0, 3.0)	0.43
Use an AED or defibrillator	2.0 (1.50, 3.0)	2.0 (1.0, 2.75)	0.27
Lead resuscitation team w/good pulse	4.0 (3.0, 4.0)	3.0 (3.0, 4.0)	0.65
Lead resuscitation team w/cardiac arrest	3.0 (2.50, 4.0)	3.0 (3.0, 3.75)	0.86
Total Likert Score	27.0 (22.50, 29.0)	23.0 (20.25, 28.75)	0.39
Post-Test			
Place IO line	4.0 (4.0, 4.50)	4.0 (3.0, 4.0)	0.43
Manage BVM ventilation	4.0 (4.0, 4.50)	4.50 (4.0, 5.0)	0.33
Intubate	4.0 (3.0, 4.0)	3.0 (2.25, 4.0)	0.50
Perform chest compressions	5.0 (4.0, 5.0)	5.0 (4.0, 5.0)	0.83
Evaluate a rhythm strip	3.0 (3.0, 4.0)	3.0 (2.25, 4.0)	0.92
Use an AED or defibrillator	3.0 (2.50, 4.0)	3.0 (2.0, 4.0)	0.83
Lead resuscitation team w/good pulse^b^	4.0 (4.0, 4.0)	4.0 (3.25, 4.75)	0.97
Lead resuscitation team w/cardiac arrest	4.0 (3.0, 4.0)	4.0 (3.0, 4.0)	0.98
Total Likert Score^b^	30.0 (27.25, 31.75)	29.0 (26.25, 34.0)	0.98

^a^ p-value was calculated using the Mann-Whitney Test

^b^ One value is missing for this variable

^c^ Likert scale: 1 = not at all confident; 2 = slightly confident, I am able to initiate the skill but would prefer assistance or direction; 3 = somewhat confident, I am able to perform the skill well but would prefer some assistance or direction; 4 = confident, I am able to perform the skill well without assistance; and 5 = very confident, I have mastered this skill and could teach it to others

Both groups rated their respective training high (very to exceptionally), with the RCDP group having a slightly higher percentage of extremely helpful compared to the traditional group (68.4% vs 61.5%, respectively); however it was not statistically significant (p-value = 0.69) due to the small sample size (results not shown).

## Discussion

Our results demonstrate that low fidelity simulation in a resource-limited setting utilizing both traditional debriefing and RCDP as instruction techniques can result in significant improvement in pediatric resident performance on simulated resuscitations. Our results also demonstrated improved self confidence in resuscitation skills in both groups. This is one of the first studies demonstrating an effective use of low-fidelity simulation to teach complex resuscitation skills in pediatric residents rather than more basic resuscitation steps such as those taught in Helping Babies Breathe or other simulation-based curricula. This is a promising finding and we hope will lead to further studies and eventual incorporation of simulation-based education into many resource-limited settings to teach complex medical management scenarios and resuscitation performance.

We did not demonstrate a difference in post-simulation performance between the RCDP and traditional simulation debriefing groups in overall performance, though this may have been due to our sample size limiting our power.

Resident self-confidence in resuscitation skills was not significantly different between the two groups but we did see a trend towards greater improvement in the RCDP group. This may reflect the increased number of opportunities to repeat skills and performance following feedback. Further study with a larger sample size is needed to better explore whether this trend is significant and whether it translates into differential performance in a clinical setting.

It is unknown whether there is a difference in the length of time it takes to achieve proficiency with teaching RCDP or traditional simulation teaching skills. As we spread this more broadly within resource-limited settings and train new simulation facilitators this could result in a significant impact on debriefing quality and resultant skill acquisition. Further study comparing results when the two techniques are facilitated by novice instructors will be critical as these simulations are more widely disseminated as a learning tool to new institutions and settings without simulation experience.

High-fidelity simulation with heightened realism has been shown to be more effective at improving performance in multiple studies [23, 24]. While our study did not compare high-fidelity or realism to our lower fidelity/realism simulation, it did demonstrate that low-fidelity simulation can result in significant improvement in even complex clinical resuscitation scenarios. High-fidelity simulators are quite expensive and cost-prohibitive for most low- and even middle-income country settings. Therefore, the demonstration that low-fidelity simulation can be an effective learning tool is valuable, and we hope will result in more studies using low-fidelity, inexpensive simulators to disseminate simulation teaching to physician trainees worldwide.

### Limitations

Our study was a single-site study with sample size limited by the number of rotating residents at CHUK during the intervention period. This was further limited by a loss of a significant number of resident performance due to technical issues involved in performing such a study in a resource-limited setting. There is no reason to think that these data were lost preferentially in one type of performance than another. There was sufficient power (100%) overall and within study groups when comparing pre and post-test scores (paired) due to the approximate 20% difference. However, when analyzing the mean percent differences between groups, there was not sufficient power (5%) due to their similarity. Future studies with a larger sample size may allow more subtle differences in performance between the two intervention groups to be detected. No validated simulation assessment tool exists for resource-limited settings. Therefore, we adapted the STAT tool to reflect changes in our setting including both available supplies and personnel. It is possible that these modifications affected the validity of the tool. However, given that the same tool was used in both groups we would expect this effect to have minimal impact on the study outcomes. We also studied ICC to evaluate the reliability of our modified tool which showed high agreement between observers. Given that residents knew that they would be tested at the end of the study period it is possible that the Hawthorne effect played a role in minimizing the difference between groups. If those who felt less prepared for post-testing studied extra because they knew a post-performance test would be administered it would minimize the effect size of one vs. the other debriefing methodology. Finally, our study is limited in that it only evaluated performance on simulated scenarios and not on participant performance on resuscitations with real patients in real emergencies. Therefore, we cannot comment on the translation of knowledge and skills from the learning environment to the clinical environment. Future studies are needed to further evaluate the impact of similar curricula on actual patient outcomes.

## Conclusions

This study demonstrates that a low-cost, low-fidelity simulation-based curriculum for pediatrics residents in Rwanda resulted in significant performance improvement in a complex simulated pediatric resuscitation. While there were trends towards more improvement in performance, resident self-confidence and satisfaction in the RCDP group we did not have the statistical power to detect whether an actual difference between these groups existed. Our results show that simulation cases utilizing instruction with both RCDP and traditioonal debriefing can be useful in teaching complex pediatric resuscitation skills that are critically needed in resource-limited settings.

## Additional files

Additional file 1: Pediatric Simulation Test Case and Modified Simulation Assessment Tool. This includes the testing case script as well as the STAT assessment tool with which the case performance was scored. (DOCX 40 kb)

Additional file 2: Modified STAT scoring guide. This is the standardized grading system by which all simulation performances were assessed on each item. (DOCX 27 kb)

Additional file 3: RCDP Respiratory Case 1. This is one of the respiratory cases we used in our RCDP simulation curriculum describing in detail the objectives and teaching points for each round. (DOCX 19 kb)

Additional file 4: Traditional Respiratory Case 1. This is the corresponding respiratory case we used in the traditional simulation curriculum outlining the objectives and teaching points for the debriefing. (DOCX 17 kb)

## Data Availability

All data generated or analyzed during this study are included in this published article [and its supplementary information files].

## Abbreviations

AED: Automated external defibrillator
ALS: Advanced life support
ANCOVA: Analysis of covariance
BVM: Bag valve mask
CHUK: Centre Hospitalier Universitaire de Kigali
CPR: Cardiopulmonary resuscitation
ICC: Intraclass correlation coefficient
IO: Intraosseous
IV: Intravenous
PGY: Post-graduate year
RCDP: Rapid cycle deliberate practice
STAT: Simulation team assessment tool
