# Supplementary material for: Rapid cycle deliberate practice vs. traditional simulation in a resource-limited setting
Source: BMC Med Educ. 2019 Aug 22;19:314. doi: 10.1186/s12909-019-1742-4 (PMC6704559; doi:10.1186/s12909-019-1742-4)
Supplement: Supplementary file 1 — Pediatric Simulation Test Case and Modified Simulation Assessment Tool. This includes the testing case script as well as the STAT assessment tool with which the case performance was scored. (DOCX 40 kb) [file 12909_2019_1742_MOESM1_ESM.docx]

**Pediatric Simulation Case**

**Instructions for Participants:**

During the next examination you will evaluate an emergency department patient while applying all the knowledge and skills you obtained so far during this training course.

Some members of your group will be assigned the role of physicians. Others will be assigned the role of nurses working in a district hospital casualty ward. You are only expected to do the tasks you are supposed to do given your professional role.

The patient you encounter for this exercise is not a real patient but a SIMULATED PATIENT. For the purpose of this evaluation, the Training Instructors prepared a mannequin and a volunteer parent to act and respond to you and your care decisions as close to a real patient encounter as you might experience in the emergency department. You can obtain all needed information from the simulated patient, as you would from a real patient.

All conversations with the simulated patient and the examiner should be in English.

Obtain any missing vital signs and demonstrate how you would perform them, ask for any additional information about the patient (signs and symptoms), and perform any necessary focused physical examinations.

Based on the patient’s chief complaint, vital signs, history, and physical exam findings, obtain any laboratory or perform medical interventions (including giving intravenous fluids and/ or medications) by stating the intervention and simulating performing it.

If you decide to administer medications, let the Course Instructor know what medication or treatment (e.g. oxygen or medications) you would like to administer. Remember, you will also need to provide the course instructor with the correct dosing for any intravenous fluids and medications you give. You cannot administer any actual medications to the mannequin, but you can apply oxygen and perform procedures such as bag-mask ventilation, intubation, and CPR if you would perform these in real life. Your orders for medications or fluids should be reported to the examiner.

All information collected during this examination will be used for evaluation of the current training program. The specifics of your examination will be kept anonymous and not connected to your name. This information will not be given to anyone else but the course evaluation team. There will be no personal repercussions to you or anyone else if your performance is imperfect.

**Pediatric Simulation Case: Respiratory Failure**

**Beginning of Case:**

An 8 month-old female is brought to your emergency department by her mother for difficulty breathing.

The mother tells you that her daughter began to have some cough and fast breathing a few days ago. Today, the child has been breathing very fast and the mother tells you it looks like it is hard for her to breathe. She is no longer able to breastfeed today. The child has also felt hot at home.

**Pediatric Simulation Case: Respiratory Failure**

**Instructions for Examiner:**

Each group of residents will be provided with the problem and chief complaint (difficulty breathing).

The group will also be provided with a short clinical vignette consisting of patient’s sex, age, and duration of problem. All information provided in the vignette will be presented clearly in order to directly lead the candidate to the simulated health problem or diagnosis.

All vital signs and exam findings will need to be requested by the candidate in order to simulate the patient encounter.

The vignette for this case is below.

**Beginning of Case:**

An 8 month-old female is brought to your emergency department by her mother for difficulty breathing.

The mother tells you that her daughter began to have some cough and fast breathing a few days ago. Today, the child has been breathing very fast and the mother tells you it looks like it is hard for her to breathe. She is no longer able to breastfeed today. The child has also felt hot at home.

**Additional Case Information for the Examiner:**

This patient presents with respiratory distress due to pneumonia.

VITALS: T 40.2 HR 190 RR 76 BP 76/38 O2 61% on RA Wt 8 kg

AIRWAY/BREATHING: She is tachypneic and hypoxic with retractions. Breath sounds are diminished on the right. Her oxygen will briefly improve with administration of oxygen, but she will have worsening work of breathing and progress to respiratory failure due to the severity of her disease. She will eventually require ventilation with BVM and intubation.

CIRCULATION: The patient is tachycardic with dry mucous membranes and few tears. She has 2+ peripheral pulses and capillary refill is 2-3 seconds.

**Pediatric Simulation Case: Respiratory Failure**

**Progression of Case for Examiners**

**Initial Assessment**

**Vitals:** T 40.2 HR 190 RR 76 BP 76/38 O2 61% on RA Wt 8 kg

**Exam:** Airway is open. Breathing with subcostal retractions, head-bobbing, nasal flaring. Lungs with crackles and decreased breath sounds to the entire right lung, with no breath sounds in the right base. Good breath sounds on the left. Circulation with 2+ peripheral pulses, tachycardic, no murmur, capillary refill 2-3 seconds. Mental status alert, briefly cries appropriately to painful stimuli. Secondary survey- Dry mucous membranes, few tears, soft abdomen, no rashes, no bruising. If asked for an exam finding not above, the finding should be reported to the student as normal.

**Team will have 3 minutes to complete general assessment, attach monitors, initiate O2, obtain IV access and order relevant studies. Labs will not return during this encounter, and x-ray is not yet available. Glucose is immediately available if requested, and is 91. After this time, team should ask for reassessment. If they do not, offer that child appears to be less alert, struggling to breathe.**

**Reassessment #1**

**Vitals:** HR 198 RR 20 BP 74/39 O2 53%

**Exam:** Airway- open. Breathing with subcostal retractions, head-bobbing, nasal flaring, grunting. Lungs with crackles and decreased breath sounds to the entire right lung, with no breath sounds in the right base. Clear on left. Circulation with 2+ peripheral pulses, tachycardic, no murmur, slightly cool extremities. Mental status AVPU: P (moans to painful stimuli). Secondary survey- Dry mucous membranes, few tears, soft abdomen, no rashes, no bruising. If asked for an exam finding not above, the finding should be reported to the student as normal.

Team may initially try to increase O2 but saturations will increase to 56% and not higher. Work of breathing will not improve. Patient will require bag-valve-mask ventilation. Saturations will improve to 92% with this if correctly done. Saturations will not improve if correct technique is not used. Team should call for intubation at this time. If ventilation is stopped, patient will desaturate. Intubation will not be successful without RSI meds as patient will have intact gag and will gag and cough.

Acceptable tube sizes: 3.5, 4.0

Acceptable RSI meds: premed- atropine, sedative- fentanyl and versed, or ketamine, paralytic- vecuronium, succinylcholine

**Decompensation during intubation**

Patient will quickly desaturate when BVM stopped for intubation and patient will arrest. Monitor will change to PEA. No waveform will be obtainable for pulse ox and no BP can be obtained. If the team does not note arrest within 2 minutes of loss of pulse, state that a student has asked why the monitor has changed.

**Reassessment after pulse check**

**Vitals:** No pulse, rhythm PEA RR rate at which bagged, no breath sounds or chest rise O2 no reading

Teams should call for AED to have it available and should connect pads, even if they correctly identify rhythm as not shockable. Teams will perform 2 rounds of CPR. After 2^nd^ round, there will be return of spontaneous circulation.

**Reassessment after ROSC**

**Vitals:** HR 198 RR rate of bagging BP 50/26 O2 95% (if bagging, drop if not bagging)

**Exam:** Airway- open if positioned and bagging, ET if previously placed. Breathing with bagging only. Lungs with crackles and decreased breath sounds to the entire right lung, with very diminished breath sounds in the right base. Clear on left. Circulation with 1+ central and peripheral pulses, tachycardic, no murmur, capillary refill 5 seconds, cool extremities. Mental status AVPU: U (unresponsive to all stimuli). Secondary survey- Pupils 4 and reactive, dry mucous membranes, soft abdomen, no rashes, no bruising. If asked for an exam finding not above, the finding should be reported to the student as normal.

If not previously secured, team should move to secure the airway at this time by intubating the patient. They should also recognize hypotension and give fluid bolus (20 mL/kg). BP will improve with fluids. Team should initiate transfer to PICU. End scenario.

**Modified Simulation Team Assessment Tool (STAT)**

Date: ______________________­­____ Study ID #:________________________

Time: __________________________

| Basics | | | | | |
| --- | --- | --- | --- | --- | --- |
| Task Group | **Task** | **Complete & Timely** | **Incomplete or Untimely** | **Needed and Not Done** | **Not Required** |
| History & Physical | Obtain SAMPLE history (sign/ symptoms, allergies, meds, past illness, last meal, events preceding) | 2 | 1 | 0 | N/A |
|  | Performs primary survey (ABCDE) | 2 | 1 | 0 | N/A |
|  | Performs secondary survey (head to toe, including back) | 2 | 1 | 0 | N/A |
| Patient Weight | Estimates/ obtains patient weight | 2 | 1 | 0 | N/A |
| Monitors | Ensures cardiorespiratory and O2 monitors placed | 2 | 1 | 0 | N/A |
| Access | Obtains or confirms vascular access | 2 | 1 | 0 | N/A |
|  | Attempts IO access | 2 | 1 | 0 | N/A |
| Labs | Orders appropriate lab testing | 2 | 1 | 0 | N/A |
| X-rays/ studies | Orders appropriate imaging | 2 | 1 | 0 | N/A |
| Recognition | Recognizes urgent/ emergent situation (either at beginning of scenario or with decompensation) | 2 | 1 | 0 | N/A |

| Airway & Breathing | | | | | |
| --- | --- | --- | --- | --- | --- |
| Task Group | **Task** | **Complete & Timely** | **Incomplete or Untimely** | **Needed and Not Done** | **Not Required** |
| Assessment | Assesses airway | 2 | 1 | 0 | N/A |
|  | Assesses breathing | 2 | 1 | 0 | N/A |
| Basic Intervention | Performs airway maneuvers | 2 | 1 | 0 | N/A |
|  | Provides supplemental oxygen | 2 | 1 | 0 | N/A |
|  | Uses appropriate adjunct airway | 2 | 1 | 0 | N/A |
| Bag-mask ventilation | Initiated BMV | 2 | 1 | 0 | N/A |
|  | Bags at appropriate rate | 2 | 1 | 0 | N/A |
|  | Assesses chest rise | 2 | 1 | 0 | N/A |
|  | Uses proper BMV technique and positioning | 2 | 1 | 0 | N/A |
| Airway RSI | Selects appropriate premed | 2 | 1 | 0 | N/A |
|  | Uses appropriate premed dose (Broselow or code sheet or dose) | 2 | 1 | 0 | N/A |
|  | Selects appropriate sedative/ induction medications | 2 | 1 | 0 | N/A |
|  | Uses appropriate sedative/ induction dose (Broselow or code sheet or dose) | 2 | 1 | 0 | N/A |
|  | Selects appropriate paralytic medication | 2 | 1 | 0 | N/A |
|  | Uses appropriate paralytic dose (Broselow or code sheet or dose) | 2 | 1 | 0 | N/A |
| Endotracheal intubation | Initiates team efforts for endotracheal intubation | 2 | 1 | 0 | N/A |
|  | Preoxygenates patient | 2 | 1 | 0 | N/A |
|  | Selects appropriate endotracheal tube size | 2 | 1 | 0 | N/A |
|  | Selects appropriate laryngoscope size | 2 | 1 | 0 | N/A |
|  | Ensures suction is on | 2 | 1 | 0 | N/A |
|  | Uses appropriate endotracheal tube insertion technique | 2 | 1 | 0 | N/A |
|  | Places endotracheal tube in trachea | 2 | 1 | 0 | N/A |
|  | Secures endotracheal tube | 2 | 1 | 0 | N/A |
| Intubation Assessment | Check end-tidal CO2 | 2 | 1 | 0 | N/A |
|  | Assesses ventilation: chest rise, auscultation | 2 | 1 | 0 | N/A |
|  | Requests chest x-ray to confirm tube placement | 2 | 1 | 0 | N/A |
| Gastric Decompression | Places NG or OG tube after intubation | 2 | 1 | 0 | N/A |

| Circulation | | | | | |
| --- | --- | --- | --- | --- | --- |
| Task Group | **Task** | **Complete & Timely** | **Incomplete or Untimely** | **Needed and Not Done** | **Not Required** |
| Basics | Assesses heart rate | 2 | 1 | 0 | N/A |
|  | Assesses pulses | 2 | 1 | 0 | N/A |
|  | Assesses blood pressure | 2 | 1 | 0 | N/A |
|  | Assesses distal perfusion (cap refill) | 2 | 1 | 0 | N/A |
| Management | Initiates volume resuscitation | 2 | 1 | 0 | N/A |
|  | Selects isotonic fluid | 2 | 1 | 0 | N/A |
|  | Initiates appropriate IV fluid dose | 2 | 1 | 0 | N/A |
|  | Ongoing fluid resuscitation as needed | 2 | 1 | 0 | N/A |
| CPR | Correct hand placement | 2 | 1 | 0 | N/A |
|  | Correct rate of compressions | 2 | 1 | 0 | N/A |
|  | Uses appropriate ratio of ventilations: compressions | 2 | 1 | 0 | N/A |
|  | Minimize interruptions in CPR | 2 | 1 | 0 | N/A |
|  | Medications (gives adrenaline appropriately) | 2 | 1 | 0 | N/A |
|  | Pulse/ rhythm check after 2 minutes | 2 | 1 | 0 | N/A |
| Arrhythmia | Recognizes abnormal rhythm | 2 | 1 | 0 | N/A |
|  | Initiates CPR | 2 | 1 | 0 | N/A |
|  | Calls for AED | 2 | 1 | 0 | N/A |
|  | Correctly places AED pads | 2 | 1 | 0 | N/A |
|  | Correctly turns on AED | 2 | 1 | 0 | N/A |

| Team Management | | | | | |
| --- | --- | --- | --- | --- | --- |
| Task Group | **Task** | **Complete & Timely** | **Incomplete or Untimely** | **Needed and Not Done** | **Not Required** |
| Team |  |  |  |  |  |
| Leadership (Team Leader) | Assigns roles to team members | 2 | 1 | 0 | N/A |
|  | Directs/ redirects team members appropriately | 2 | 1 | 0 | N/A |
|  | Uses closed-loop communication (orders directed & confirmed) | 2 | 1 | 0 | N/A |
|  | Maintains global view (does not get sidetracked by procedures, details) | 2 | 1 | 0 | N/A |
|  | Performs tasks in appropriate sequence/ prioritizes well | 2 | 1 | 0 | N/A |
|  | Reprioritizes for urgent/ emergent events | 2 | 1 | 0 | N/A |
|  | Avoids fixation errors (considers full differential for problems encountered) | 2 | 1 | 0 | N/A |
|  | Performs interim summary/ assessment for team coordination | 2 | 1 | 0 | N/A |
|  | Work-load balancing | 2 | 1 | 0 | N/A |

**Timed Metrics**

Time from start of scenario to O2: _____________________________________________________

Time from start of scenario to BVM: ____________________________________________________

Time from loss of pulse to recognition of pulselessness: ____________________________________

Time from recognition of pulselessness to call for AED: _____________________________________

Time from recognition of pulselessness to attaching AED: ___________________________________

Time from recognition of pulselessness to compressions: ___________________________________
