# Supplementary material for: Rapid cycle deliberate practice vs. traditional simulation in a resource-limited setting
Source: BMC Med Educ. 2019 Aug 22;19:314. doi: 10.1186/s12909-019-1742-4 (PMC6704559; doi:10.1186/s12909-019-1742-4)
Supplement: Supplementary file 2 — Modified STAT scoring guide. This is the standardized grading system by which all simulation performances were assessed on each item. (DOCX 27 kb) [file 12909_2019_1742_MOESM2_ESM.docx]

| BASICS | | | | | |
| --- | --- | --- | --- | --- | --- |
| Task Group | **Task** | **2 points** | **1 point** | **0 points** | **Not applicable** |
| History & Physical | Solicits SAMPLE history (signs/ symptoms, allergies, meds, past illness, last meal, events preceding) | - Obtains key relevant additional historical elements | - Obtains incomplete information - Gathers information in an untimely fashion | - Not done | - Not needed in scenario |
|  | Performs primary survey (ABCDE) | - Completes in first 2 minutes | - Completes after first 2 minutes - Starts, but does not complete | - Not done | - Not needed in scenario |
|  | Performs secondary survey (head to toe exam, including back) | - Completes in first 8 minutes | - Completes secondary survey after first 8 minutes - Starts, but does not complete | - Not done | - Not needed in scenario |
| Patient Weight | Estimates/ obtains pt weight | - Obtains within 2 minutes - Uses scale or size-based tape - Obtains from current chart | - Obtains after 2 min- when meds are given - Estimates weight based on size/ age - Asks parent for recent weight | - Not done | - Not needed in scenario |
| Monitors | Ensures cardiorespiratory and O2 monitors placed | - Completes within first 60 seconds | - Completes >60 seconds - Forgets one monitor | - Not done | - Not needed in scenario |
| Access | Obtains vascular access | - Completes within first 60 seconds | - Done in > 60 seconds | - Not done | - Not needed in scenario |
|  | Attempts IO access | - If no IV access after 2 attempts or >2 min | - Done but after >2 IV attempts - Done but after > 90 seconds with IV attempts | - Not done | - Not needed in scenario |
| Labs | Orders appropriate lab testing | - Orders labs within first 5 minutes | - Orders labs after 5 minutes, resulting in diagnosis or treatment delay | - Not done | - Not needed in scenario |
| X-rays/ studies | Orders appropriate imaging | - Orders in timely fashion - Orders CXR after intubation | - Orders delayed | - Not done | - Not needed in scenario |
| Recognition | Recognizes urgent/ emergent situation (either start of scenario or with decompensation) | - <30 seconds from start of scenario or acute decompensation - Recognizes need for full code response | - Responds slowly to emergent situation (>30 sec but <2 min) | - Not done - >2 min | - Not needed in scenario |
| AIRWAY/BREATHING | | | | | |
| Task Group | **Task** | **2 points** | **1 point** | **0 points** | **Not applicable** |
| Assessment | Assesses airway | - Checks patency: looks, listens, feels - Part of primary survey and with acute decompensation | - Assesses during primary survey or decompensation but not both | - Not done | - Not needed in scenario |
|  | Assesses breathing | - All of the following: - Looks for chest rise - Auscultates both sides - Counted or reviewed RR on monitor - Assesses during primary survey | - Only one of the previous column - Auscultates one side of chest - Assesses during primary survey or decompensation but not both | - Not done | - Not needed in scenario |
| Basic intervention | Performs airway maneuvers | - Provides jaw thrust/ chin lift - Places shoulder roll or repositions pt to open airway | - Airway incompletely open/ maintained | - Not done - If trauma pt, uses jaw thrust | - Not needed in scenario |
|  | Provides supplemental oxygen | - 100% oxygen provided - Maximized oxygen delivery (face mask, NRB) | - Uses <100% oxygen source - Uses blow by oxygen only | - Not done | - Not needed in scenario |
|  | Uses appropriate adjunct airway | - Nasal airway if conscious/ gag intact (airway not patent despite positioning alone) - Oral airway if unconscious/ no gag (airway not patent despite positioning alone | - Uses oral airway in conscious patient | - Not done | - Not needed in scenario |
| Bag-mask ventilation | Initiates BMV | - Recognizes need for respiratory support <30 seconds from onset - Ensures self-inflating bag hooked up to oxygen | - Recognizes need for respiratory support but >30 sec and <2 min - Does not connect oxygen | - Not done - Recognizes need for support >2 min | - Not needed in scenario |
|  | Bags at appropriate rate | - Bags at age appropriate rate - Allows time for exhalation - Volume approximately 10 mL/kg/breath | - Bags at RR < or > appropriate - Volume < or > | - Not done | - Not needed in scenario |
|  | Assesses chest rise | - Visually or verbally confirms chest rise with ventilation | - Auscultates breath sounds | - Not done | - Not needed in scenario |
|  | Uses proper BMV technique and positioning | - Mask fit over nose and mouth - Mask does not touch eyes - C&E hand positioning - Good seal | - Incomplete mask fit - Hand positioning leads to incomplete seal or airway obstruction | - Not done | - Not needed in scenario |
| Airway RSI | Selects appropriate premed | - Uses appropriate premeds: atropine <5 year, lidocaine for ICP, fentanyl for pain | - Premed selection incomplete | - Not done | - Not needed in scenario |
|  | Uses appropriate premed dose | - Orders correct dose and correct dose reaches pt | - Incorrect dose ordered but corrected and correct dose reaches pt | - Orders incorrect dose and incorrect dose reaches pt | - Not needed in scenario |
|  | Selects appropriate sedative/ induction medications | - Selects best RSI sedative/ induction agent for situation | - Selects non-RSI sedative - Sedative used not best for situation | - Not done - Contra-indicated med given | - Not needed in scenario |
|  | Uses appropriate sedative/ induction dose | - Orders correct dose and correct dose reaches pt | - Incorrect dose ordered but corrected and correct dose reaches pt | - Orders incorrect dose and incorrect dose reaches pt | - Not needed in scenario |
|  | Selects appropriate paralytic | - Selects RSI paralytic | - Selects non-RSI paralytic (delayed onset) | - Not done | - Not needed in scenario |
|  | Uses appropriate paralytic dose | - Orders correct dose and correct dose reaches pt | - Incorrect dose ordered but corrected and correct dose reaches pt | - Orders incorrect dose and incorrect dose reaches pt | - Not needed in scenario |
| Endotracheal intubation | Initiates team efforts for endotracheal intubation | - Orders team to prepare for intubation - Confirms O2, suction, equipment, and personnel preparedness - Assigns team members to cricoid, equipment assistance and confirmation of placement | - Incomplete or delayed preparation, coordination, or assessment | - Not done | - Not needed in scenario |
|  | Pre-oxygenates patient | - NRB or BMV with 100% O2 | - Face mask or blow-by with 100% oxygen | - Not done | - Not needed in scenario |
|  | Selects appropriate endotracheal tube size | - Uses age appropriate size: 4+ (age in years/4) or Broselow tape size - +/- 0.5 size tube | - +/- 1.0 in size | - Greater or less than 1.0 in size | - Not needed in scenario |
|  | Selects appropriate laryngoscope size | - Uses age appropriate Mac or Miller size | - +/- 1 Miller/ Mac | - > +/- 1 Miller/ Mac | - Not needed in scenario |
|  | Ensures suction is on | - Turns Yankauer/ wide suction on | - Assembles but does not turn on suction | - Not done | - Not needed in scenario |
|  | Uses appropriate endotracheal tube insertion technique | - Uses stylet - Achieves jaw lift without dental impact or c-spine flexion | - Does not use stylet - Dental impact - C-spine flexes | - Not done | - Not needed in scenario |
|  | Places endotracheal tube in trachea | - Places ETT in trachea, above the bifurcation | - Intubates right or left mainstem, not identified and corrected | - Not done - ETT not in airway | - Not needed in scenario |
|  | Endotracheal tube secured | - Secures ETT for transport/ ongoing care with tape, holder | - Secures inadequately - Held in place by hand | - Not done | - Not needed in scenario |
| Intubation assessment | Checks end-tidal CO2 | - Immediately uses pedi-cap or end tidal CO2 monitor after intubation | - Delays use of pedi-cap or end-tidal CO2 monitor | - Not done | - Not needed in scenario |
|  | Assesses ventilation: chest rise, auscultation | - Assesses chest rise and auscultates bilaterally | - Fails to assess chest rise or auscultates only unilaterally | - Not done | - Not needed in scenario |
|  | Requests portable chest x-ray to confirm tube placement | - Orders CXR <60 sec after intubation | - Orders CXR but >60 sec after intubation | - Not done | - Not needed in scenario |
| Gastric decompression | Places NG or OG after intubation | - Places NG or OG <60 seconds after intubation (if not done previously) | - Places NG or OG >60 seconds after intubation | - Not done | - Not needed in scenario |

| CIRCULATION | | | | | |
| --- | --- | --- | --- | --- | --- |
| Task group | **Task** | **2 points** | **1 point** | **0 points** | **Not applicable** |
| Basics | Assesses heart rate | - Auscultates heart rate - Notes HR on monitor with primary survey and acute decompensation | - Assesses during primary survey or decompensation but not both | - Not done | - Not needed in scenario |
|  | Assesses pulses | - Assesses central (brachial or femoral) pulse - Assesses peripheral pulses - During primary survey and acute decompensation | - Assesses only peripheral pulses - Assesses carotid pulse - Primary survey or decompensation but not both | - Not done | - Not needed in scenario |
|  | Assesses blood pressure | - Notes BP on monitor - Performs manual BP - Assesses during primary survey and acute decompensation | - Assesses during primary survey or acute decompensation but not both | - Not done | - Not needed in scenario |
|  | Assesses distal perfusion | - Checks distal capillary refill | - Checks with primary survey or decompensation but not both | - Not done | - Not needed in scenario |
| Management | Initiates volume resuscitation | - If in shock, started within 2 min | - If in shock, started after 2 min | - Not done | - Not needed in scenario |
|  | Selects isotonic fluid | - Uses NS or LR for bolus |  | - Not done - Bolused with non-isotonic fluid | - Not needed in scenario |
|  | Initiates appropriate IV fluid dose | - 20 mL/kg fluid bolus if no cardiac concerns voiced - 10 mL/kg fluid bolus if voiced cardiac concerns | - <20 mL/kg fluid bolus if no cardiac concerns voiced | - Not done | - Not needed in scenario |
|  | Ongoing fluid resuscitation as needed | - Additional 20 mL/kg fluid boluses given for persistent symptoms in a timely fashion | - Additional fluid boluses <20 mL/kg or slow to be given | - No additional fluids given, but more needed in scenario | - Not needed in scenario |
| CPR | Correct hand placement | - All providers correctly place hands for giving CPR | - Hand placement initially incorrect, but fixed by one or more providers | - Not done - Hand placement incorrect, not fixed by one or more providers | - Not needed in scenario |
|  | Correct rate of compressions | - All providers deliver compressions at a rate of > or = to 100 bpm | - One or more providers deliver compressions <100 or >150, but adjust | - Not done - Chest compressions <100 bpm, not fixed | - Not needed in scenario |
|  | Uses appropriate surface (backboard, floor) | - Places backboard under patient - Places pt on hard surface | - Initially not used but eventually used | - Not done | - Not needed in scenario |
|  | Uses appropriate ventilation: compression ratio | - 15:2 if bag-valve mask - Unsynched if intubated | - Initially not using appropriate ratio but fixed | - Not done | - Not needed in scenario |
|  | Minimizes interruptions in CPR | - No interruptions | - Minimal, but occasional, interruptions | - Prolonged interruptions | - Not needed in scenario |
|  | Medications (gives adrenaline appropriately) | - Gives adrenaline at correct point in algorithm and correct dose | - Gives adrenaline >2 minutes into scenario but gives correct dose | - Does not give adrenaline when needed or gives incorrect dose | - Not needed in scenario |
|  | Pulse/rhythm check after 2 minutes | - Checks both rhythm and pulse after CPR for 5 cycles | - Does not check pulse OR rhythm | - Not done | - Not needed in scenario |
|  |  |  |  |  |  |
| Arrhythmia | Recognizes abnormal rhythm | - <30 sec from onset - Identifies arrhythmia correctly | - >30 sec but <2 min - Incorrectly identifies arrhythmia | - >2 min - Not done | - Not needed in scenario |
|  | Initiates CPR | - <30 sec after recognizes pulselessness | - Does but without pulse check - Recognizes >30 sec but <2 min after pulselessness | - Not done - >2 min | - Not needed in scenario |
|  | Calls for AED | - <30 sec from onset | - >30 sec but <2 min | - >2 min - Not done | - Not needed in scenario |
|  | Correctly places AED pads | - Places pads correctly | - Initially placed incorrectly but fixed | - Not done - Places incorrectly | - Not needed in scenario |
|  | Correctly turns on AED | - Turns on AED immediately | - Delay in turning on AED >30 sec but <2 minutes from arrival at bedside | - Not done | - Not needed in scenario |

| TEAM MANAGEMENT | | | | | |
| --- | --- | --- | --- | --- | --- |
| Task group | **Task** | **2 points** | **1 point** | **0 points** | **Not applicable** |
|  | Assigns roles to team members | - Team leader verbally/ visually assigns team members roles | - Assigns some team members roles verbally or visually | - Not done | - Not needed in scenario |
|  | Directs/ redirects team members appropriately | - Gives clear verbal orders - Prioritizes orders clearly for team - If team members raise finding/ ask questions, team leader provides direction for team to prioritize response | - Team on 2 or less occasions raises concerns/ questions/ issues not addressed by team leader resulting in team not acting cohesively | - Not done | - Not needed in scenario |
|  | Uses closed loop communication (orders directed and confirmed) | - Consistently directs orders to specific individuals, requested confirmation when task complete | - Intermittently uses closed loop communication | - Not done | - Not needed in scenario |
|  | Maintains global view (does not get sidetracked) | - Monitors overall situation, while aware of details - Turns out unnecessary details | - Recognizes big picture - Tunes out majority of unnecessary details | - Fails to see “big picture” - Distractible, unable to tune out unnecessary details | - Not needed in scenario |
|  | Performs tasks in appropriate sequence/ prioritizes well | - Addresses ABCDE’s in orderly fashion - Prioritizes interventions | - Orderly approach to patient most of the time - Occasionally, delays in most urgent priority but without significantly impacting patient care | - Disorderly approach to patient care - Delays in urgent interventions, negatively impacting patient care | - Not needed in scenario |
|  | Reprioritizes for urgent/ emergent event | - Readily recognizes changes in patient condition and directs team to intervene appropriately | - Recognizes changes in condition readily most of the time - Delays or ineffectively redirects team | - Recognizes emergent change in patient status slowly - Fails to redirect team to emergent issues | - Not needed in scenario |
|  | Avoids fixation errors (considers full differential) | - Does not prematurely narrow differential diagnosis | - Briefly narrows differential diagnosis, but is able to widen differential diagnosis without delay in harming patient | - Prematurely focuses on specific diagnosis, negatively impacting care | - Not needed in scenario |
|  | Provides interim summary/ assessment for team coordination | - Succinctly defines medical issues - Speaks clearly - Clearly addresses questions | - Identifies problem, but may not state clearly - Difficult to hear or understand - Does not address/ resolve questions/ concerns effectively | - Identified problem, but may not state clearly - Difficult to hear or understand - Does not address/ resolve question | - Not needed in scenario |
|  | Workload balancing | - Recognizes when specific roles over-extended, reassigns or assists in task redistribution | - Delegates work load appropriately, but does not utilize all to the best of their ability | - Fails to redistribute workload when member overextended | - Not needed in scenario |
