# Supplementary material for: Rapid cycle deliberate practice vs. traditional simulation in a resource-limited setting
Source: BMC Med Educ. 2019 Aug 22;19:314. doi: 10.1186/s12909-019-1742-4 (PMC6704559; doi:10.1186/s12909-019-1742-4)
Supplement: Supplementary file 3 — RCDP Respiratory Case 1. This is one of the respiratory cases we used in our RCDP simulation curriculum describing in detail the objectives and teaching points for each round. (DOCX 19 kb) [file 12909_2019_1742_MOESM3_ESM.docx]

**RCDP Respiratory Case 1: Respiratory Distress Requiring Intubation**

**Timeframe for each cycle:** 10, 10, 15, 15, 15

**Room setup for each round:** Infant on bed, no monitors connected, no IV or IO in room. Equipment to side of bed (monitors, IV/IO, O2, BVM, intubation equipment, meds)

**Common prompt for each cycle:** “A nurse has just called you to the emergency room to assess a patient. The patient is a 4 month male, previously healthy, with respiratory distress for 2 days. The nurse found him to be unresponsive on arrival.”

**For each round in addition to specific objectives incorporate teamwork objectives where appropriate:**

- - Closed loop communication
  - Role assignment
  - Direction of team members
  - Prioritization of tasks
  - Interim summary
  - Work-load balancing

_____________________________________________________________________________________

**Act 1: Respiratory distress/ needs O2**

**Objectives in this act**

1. Rapid assessment of CAB, recognize respiratory distress in a patient with a pulse

2. Position airway in patient with respiratory distress

3. Placement of 100% O2 for hypoxic patient in distress

4. Place monitors and attempt IV access

**Vital signs:** T 39.0 HR 180 RR 80 BP 82/43 O2 70% on RA

**Physical exam findings** (if asked): subcostal retractions, nasal flaring, crackles heard on auscultation of right lung

**Expected actions after verbal prompt:**

- Rapid assessment of pulses (femoral or brachial on infant)
- Pulses should be found
- Move to assessment of airway/ breathing, recognize respiratory distress
- Position airway
- Place O2, 100% NRB
- Ask second provider to connect monitors, attempt IV access

**Progression of scenario:**

If airway positioned and 100% O2 applied, pt with improvement of saturations to 95%, RR improves

**END:**

- If airway positioned and 100% O2 applied, give feedback and advance to Act 2
- If effective BMV given as well with proper technique, give feedback and advance to Act 3

_____________________________________________________________________________________

**Act 2: Respiratory failure/ needs BMV**

**Objectives in this act**

1. Rapid assessment of CAB, recognize apnea in a patient with a pulse

2. Position airway in patient with apnea and recognize need for bag-valve mask ventilation

- Action-linked phrase: “The patient is not breathing, I am opening the airway”

- Head tilt, chin lift or jaw thrust

3. Choose correct bag and mask, connect to O2

- Infant or pediatric bag, mask to cover nose and mouth

- Connect to O2, 15 LPM

4. Use correct technique for 1 and 2 person BMV

-1 person: C-E technique to hold mask

-2 person: 2 handed seal for first provider, second rescuer delivers breaths

5. Correct rate for ventilation

-Insufflation over 1 second, breaths given every 3-5 seconds

-Assess for effective ventilation (chest rise, auscultation)

**Vital signs:** T 39.0 HR 180 RR 0 BP 82/43 O2 50%

**Physical exam findings** (if asked): unresponsive, no chest movement, no air entry

**Expected actions after verbal prompt:**

- Rapid assessment of pulses (femoral or brachial on infant)
- Pulses should be found
- Move to assessment of airway/ breathing, recognize apnea
- Position airway
- Begin bag-valve mask ventilation
- Ask second provider to connect monitors, attempt IV access

**Progression of scenario:**

If airway positioned and effective BMV, saturations improve to 100%

**END:**

- If effective BMV given as well with proper technique, give feedback and advance to Act 3

_____________________________________________________________________________________

**Act 3: Respiratory failure/ prepare for intubation**

**Objectives in this act**

1. Recognize apnea and need for intubation

2. Prepare equipment for intubation

- Suction
- Bag-valve mask (should be bagging prior to intubation)
- Correct size for ETT
- Correct blade

3. Select medications for intubation

- Pre-medication: Atropine 0.01 mg/kg in children <2
- Sedation: Midazolam 0.1-0.2 mg/kg, Morphine 0.1-0.2 mg/kg, Fentanyl 1-2 mcg/kg, ketamine 1-2 mg/kg
- Paralytic: Succinylcholine 1 mg/kg, Vecuronium 0.1 mg/kg

**Vital signs:** T 39.0 HR 180 RR 0 BP 82/43 O2 50%

**Physical exam findings** (if asked): unresponsive, no chest movement, no air entry

**Expected actions after verbal prompt:**

- Rapid assessment of pulses (femoral or brachial on infant)
- Pulses should be found
- Move to assessment of airway/ breathing, recognize apnea
- Position airway
- Begin bag-valve mask ventilation
- Ask second provider to connect monitors, attempt IV access
- Recognize need for ongoing ventilation, call for intubation
- Collect necessary supplies and order medications

**Progression of scenario:**

If airway positioned and effective BMV, saturations improve to 100%. Patient will desat if ventilation stopped. Team will need to ask for intubation supplies.

**END:**

- If effective BMV, recognizes need for intubation and collects supplies, give feedback and advance to Act 4

_____________________________________________________________________________________

**Act 4: Respiratory failure/ intubate**

**Objectives in this act**

1. Ensure correct equipment available

2. Correct medication administration

- Pre-meds first, then sedation, then paralysis

3. Recognize when patient paralyzed

4. Ensure adequate pre-oxygenation prior to attempting intubation

5. Correct intubation technique

- Blade in left hand
- Enter right side of mouth
- Locate cords
- Do not look away from cords once visualized
- Place tube

**Vital signs:** T 39.0 HR 180 RR 0 BP 82/43 O2 50%

**Physical exam findings** (if asked): unresponsive, no chest movement, no air entry

**Expected actions after verbal prompt:**

- Rapid assessment of pulses (femoral or brachial on infant)
- Pulses should be found
- Move to assessment of airway/ breathing, recognize apnea
- Position airway
- Begin bag-valve mask ventilation
- Ask second provider to connect monitors, attempt IV access
- Recognize need for ongoing ventilation, call for intubation
- Collect necessary supplies and order medications
- Give medications
- Intubate patient once paralyzed

**Progression of scenario:**

If airway positioned and effective BMV, saturations improve to 100%. Patient will desaturate if ventilation stopped. Team will need to recognize need for intubation. If correct meds given, patient will be paralyzed for intubation. 30 seconds to intubate, if not intubated within that time, drop O2 saturations.

**END:**

- If successful intubation, give feedback and progress to Act 5

_____________________________________________________________________________________

**Act 5: Respiratory failure/ intubate & confirm tube placement, secure tube**

**Objectives in this act**

1. Confirm tube is endotracheal

Mist in tube

Chest rise

Auscultation

O2 saturations

Recognize that if capnography were available, should be used

2. Assess for proper depth

Estimate: 3x ETT diameter

Auscultate for symmetric air movement

3. Secure tube

Do not let go of tube until taped

Note depth prior to taping

4. Call for chest x-ray

**Vital signs:** T 39.0 HR 180 RR 0 BP 82/43 O2 50%

**Physical exam findings** (if asked): unresponsive, no chest movement, no air entry

**Expected actions after verbal prompt:**

- Rapid assessment of pulses (femoral or brachial on infant)
- Pulses should be found
- Move to assessment of airway/ breathing, recognize apnea
- Position airway
- Begin bag-valve mask ventilation
- Ask second provider to connect monitors, attempt IV access
- Recognize need for ongoing ventilation, call for intubation
- Collect necessary supplies and order medications
- Give medications
- Intubate patient once paralyzed
- Confirm tube is endotracheal
- Secure tube
- Call for CXR

**Progression of scenario:**

If airway positioned and effective BMV, saturations improve to 100%. Patient will desataturate if ventilation stopped. Team will need to recognize need for intubation. If correct meds given, patient will be paralyzed for intubation. 30 seconds to intubate, if not intubated within that time, drop O2 saturations. Team should secure tube and call for x-ray

**END:**

- If successful intubation and tube secured, give feedback and end scenario
