# Supplementary material for: Rapid cycle deliberate practice vs. traditional simulation in a resource-limited setting
Source: BMC Med Educ. 2019 Aug 22;19:314. doi: 10.1186/s12909-019-1742-4 (PMC6704559; doi:10.1186/s12909-019-1742-4)
Supplement: Supplementary file 4 — Traditional Respiratory Case 1. This is the corresponding respiratory case we used in the traditional simulation curriculum outlining the objectives and teaching points for the debriefing. (DOCX 17 kb) [file 12909_2019_1742_MOESM4_ESM.docx]

**Traditional Respiratory Case 1: Respiratory Distress Requiring Intubation**

**Room setup:** Infant on bed, no monitors connected, no IV or IO in room. Equipment to side of bed (monitors, IV/IO, O2, BVM, intubation equipment, meds)

**Common prompt for each cycle:** “A nurse has just called you to the emergency room to assess a patient. The patient is a 4 month male, previously healthy, with respiratory distress for 2 days. The nurse found him to be unresponsive on arrival.”

_____________________________________________________________________________________

**Vital signs:** T 39.0 HR 180 RR 0 BP 82/43 O2 50% on RA

**Physical exam findings** (if asked): unresponsive, no chest movement, no air entry

**Expected actions after verbal prompt:**

1. Rapid assessment

- Are you okay/ stimulation
- C-A-B

2. Proper bag-valve mask ventilation

- Choose correct bag and mask, connect to O2
  - Infant or pediatric bag, mask to cover nose and mouth
  - Connect to O2, 15 LPM
- Use correct technique for 1 and 2 person BMV
  - -1 person: C-E technique to hold mask
  - -2 person: 2 handed seal for first provider, second rescuer delivers breaths
- Correct rate for ventilation
  - -Insufflation over 1 second, breaths given every 3-5 seconds
  - -Assess for effective ventilation (chest rise, auscultation)

3. Intubation Equipment

- Prepare equipment for intubation
  - Suction
  - Bag-valve mask (should be bagging prior to intubation)
  - Correct size for ETT
  - Correct blade
- Select medications for intubation
  - Pre-medication: Atropine 0.01 mg/kg in children <2, minimum dose 0.1 mg
  - Sedation: Midazolam 0.1-0.2 mg/kg, Morphine 0.1-0.2 mg/kg, Fentanyl 1-2 mcg/kg, ketamine 1-2 mg/kg
  - Paralytic: Succinylcholine 1 mg/kg, Vecuronium 0.1 mg/kg
  - Use Broselow, Harriet Lane, resuscitation card when needed for doses

4. Intubation

- Pre-meds first, then sedation, then paralysis
- Recognize when patient paralyzed
- Ensure adequate pre-oxygenation prior to attempting intubation
- Correct intubation technique
  - Blade in left hand
  - Enter right side of mouth
  - Locate cords
  - Do not look away from cords once visualized
  - Place tube

5. Assessment of tube placement and securing tube

- Confirm tube is endotracheal
  - Mist in tube
  - Chest rise
  - Auscultation
  - O2 saturations
  - Recognize that if capnography were available, should be used
- Assess for proper depth
  - Estimate: 3x ETT diameter
  - Auscultate for symmetric air movement
- Secure tube
  - Do not let go of tube until taped
  - Note depth prior to taping
- Call for chest x-ray if available
- Call PICU
- Teamwork
  - Closed loop communication
  - Role assignment
  - Direction of team members
  - Prioritization of tasks
  - Interim summary
  - Work-load balancing

**Progression of scenario:**

If airway positioned and effective BMV, saturations improve to 100%. Patient will desaturate if ventilation stopped. Team will need to recognize need for intubation. If correct meds given, patient will be paralyzed for intubation. 30 seconds to intubate, if not intubated within that time, drop O2 saturations. Team should secure tube and obtain CXR.

**END:**

After intubation leave 3-5 minutes to assess placement, secure tube, call for x-ray and PICU. End scenario and give feedback.
